# *Anopheles gambiae* larvae mount stronger immune responses against bacterial infection than adults: evidence of adaptive decoupling in mosquitoes

**DOI:** 10.1186/s13071-017-2302-6

**Published:** 2017-08-01

**Authors:** Garrett P. League, Tania Y. Estévez-Lao, Yan Yan, Valeria A. Garcia-Lopez, Julián F. Hillyer

**Affiliations:** Department of Biological Sciences, Vanderbilt University, Nashville, TN USA

**Keywords:** Diptera, Hemocyte, Phagocytosis, Melanization, Phenoloxidase, Antimicrobial peptide, Hemolymph, Metamorphosis

## Abstract

**Background:**

The immune system of adult mosquitoes has received significant attention because of the ability of females to vector disease-causing pathogens while ingesting blood meals. However, few studies have focused on the immune system of larvae, which, we hypothesize, is highly robust due to the high density and diversity of microorganisms that larvae encounter in their aquatic environments and the strong selection pressures at work in the larval stage to ensure survival to reproductive maturity. Here, we surveyed a broad range of cellular and humoral immune parameters in larvae of the malaria mosquito, *Anopheles gambiae*, and compared their potency to that of newly-emerged adults and older adults.

**Results:**

We found that larvae kill bacteria in their hemocoel with equal or greater efficiency compared to newly-emerged adults, and that antibacterial ability declines further with adult age, indicative of senescence. This phenotype correlates with more circulating hemocytes and a differing spatial arrangement of sessile hemocytes in larvae relative to adults, as well as with the individual hemocytes of adults carrying a greater phagocytic burden. The hemolymph of larvae also possesses markedly stronger antibacterial lytic and melanization activity than the hemolymph of adults. Finally, infection induces a stronger transcriptional upregulation of immunity genes in larvae than in adults, including differences in the immunity genes that are regulated.

**Conclusions:**

These results demonstrate that immunity is strongest in larvae and declines after metamorphosis and with adult age, and suggest that adaptive decoupling, or the independent evolution of larval and adult traits made possible by metamorphosis, has occurred in the mosquito lineage.

**Electronic supplementary material:**

The online version of this article (doi:10.1186/s13071-017-2302-6) contains supplementary material, which is available to authorized users.

## Background

Mosquitoes face the threat of infection during all stages of their holometabolous life cycle. These threats arise from numerous sources, including the microbe-rich aquatic environments where larvae reside and the infected blood meals that adults ingest. To combat pathogens that invade their hemocoel (body cavity), mosquitoes have evolved a diverse array of cellular and humoral immune responses [1]. These responses begin when pathogen-associated molecular patterns are recognized by host-derived pattern recognition receptors. This recognition triggers the amplification or repression of extracellular signaling cascades, the initiation of intracellular signal transduction pathways and, ultimately, the activation or expression of immune effectors. These immune effectors, together with the circulating and sessile hemocytes (insect blood cells) that produce many of them, kill pathogens via phagocytosis, lysis, melanization, and other mechanisms [2, 3].

Despite the critical importance of immune responses for survival, the vast majority of what we know about the mosquito immune system comes exclusively from experiments conducted on adult females, as this stage and sex is responsible for the transmission of blood-borne pathogens such as those that cause dengue [4–6], lymphatic filariasis [7, 8], and malaria [9, 10]. Recently, we described the functional integration of the circulatory and immune systems of mosquito larvae, whereby pathogens are phagocytosed and melanized by tracheal tuft hemocytes that surround the sole entry point of hemolymph (insect blood) into the heart [11]. However, few other studies have described immune responses in larvae. Most of these studies have focused on uninfected larvae by assaying the developmental expression of immunity genes [12–25] or by describing hemocyte subpopulations [26, 27]. Few studies describe immune response in larvae assessed immune gene expression or protein levels in infected larvae [28–32], and prior to our recently published work [11], to our knowledge only one study had described a larval immune response: the encapsulation of the aquatic fungus, *Lagenidium giganteum*, by *Aedes aegypti* [33].

Although the immune system of mosquito larvae remains largely unexplored, two general lines of reasoning led us to hypothesize that larvae have evolved more proficient means of neutralizing infections than adults. First, by virtue of inhabiting aquatic environments that are rife with microorganisms, mosquito larvae are under constant threat of infection, whereas adults are less likely to encounter pathogens in their terrestrial and aerial habitats. Secondly, larvae have yet to fulfill any of their reproductive potential, and hence, strong selection pressures must be at work on the larval immune system to increase the probability of their survival to reproductive maturity [34–36]. As a corollary to this, as mosquito adults age and fulfill their reproductive potential, investment in the immune system is expected to wane in order to reallocate resources to other adult processes, such as reproduction [37–39]. If larval and adult immune responses indeed differ in either strength or composition, this would imply that future studies could no longer assume complete continuity in immune responses across life stages, and that metamorphosis has, to some extent, decoupled the larval and adult immune systems, thus enabling their independent evolution [40, 41]. Furthermore, if immune responses differ across life stages, this would have important implications for the creation of stage-specific control measures that are better tailored to the specific immune responses of each life stage.

To test the hypothesis that larvae display stronger immune responses compared to adults, and to gain a better mechanistic understanding of the larval response to infection, we conducted a series of comparative analyses of immune responses in larval and adult *Anopheles gambiae*. We show that mosquito larvae are more proficient than adults in killing bacteria, and that this correlates with stronger cellular and humoral immune responses in larvae compared to adults. Furthermore, we uncovered evidence of immune senescence within the adult stage, as the antimicrobial immune response in the hemocoel of adults declines over the first five days of adulthood.

## Methods

### Mosquito rearing and maintenance

*Anopheles gambiae* Giles (*sensu stricto*; G3 strain) were reared and maintained at 27 °C and 75% relative humidity in an environmental chamber set to a 12:12 h light:dark cycle. Larvae were reared in plastic tubs containing deionized water and fed a combination of koi food and baker’s yeast. The resulting pupae were separated from the larvae and placed into plastic containers with marquisette tops, and adults that emerged were fed a 10% sucrose solution. All experiments were performed on fourth-instar larvae and adult female mosquitoes at 1 and 5 days post-emergence.

### Mosquito injection and bacterial infection

Mosquito larvae were immobilized by removal of excess water and injected at the mesothorax [42]. Mosquito adults were cold anesthetized and injected at the thoracic anepisternal cleft. For all experiments involving bacterial injection, a Nanoject II Auto-Nanoliter Injector (Drummond Scientific Company, Broomall, PA, USA) was used to inject 69 nl of the following: live, tetracycline resistant, GFP-expressing *E. coli* (modified DH5α; GFP-*E. coli*) in Luria-Bertani’s rich nutrient medium (LB), heat-killed GFP-*E. coli*, heat-killed *Micrococcus luteus*, heat-killed *Enterobacter* sp. isolate Ag1 [43, 44], or LB medium alone. For all experiments involving cellular staining solutions (see below), approximately 0.2 μl of a solution was injected using an aspirator.

Bacteria were grown overnight in a 37 °C (or 27 °C for *Enterobacter*) shaking incubator until they reached an optical density of approximately OD_600_ = 5, as measured in a BioPhotometer plus spectrophotometer (Eppendorf AG, Hamburg, Germany). For experiments using live GFP-*E. coli*, live bacteria were injected at either OD_600_ = 5, which we refer to as the high dose, or diluted with LB medium to OD_600_ = 1, which we refer to as the low dose. Unless otherwise stated, all experiments were carried out at the high dose. The absolute doses of the bacterial inoculums were determined by performing serial dilutions of the GFP-*E. coli* cultures and spreading them on LB agar plates. These plates were grown overnight at 37 °C, the number of resulting colony forming units (CFUs) were counted, and the doses were calculated. Low doses of *E. coli* (OD_600_ = 1) ranged from 1932 to 33,741 (average = 18,061) and high doses (OD_600_ = 5) ranged from 53,889 to 77,970 (average = 65,171). These high intensity doses were selected because the pathogenicity associated with an *E. coli* infection is low relative to infection with other bacteria [45]. For experiments using heat-killed bacteria, 50 μl of bacterial culture was incubated at 95 °C for 10 min on an IncuBlock heating block (Denville Scientific, Holliston, MA, USA), and injected after cooling. Plating of the heat-killed cultures, which resulted in no CFUs, confirmed that all bacteria were dead.

### Measurement of in vivo bacteria killing efficiency

Mosquito larvae, 1-day-old adults and 5-day-old adults were injected with either low or high doses of GFP-*E. coli*. After 24 h, mosquitoes were homogenized in phosphate buffered saline (PBS; pH 7.0) and a dilution was spread on LB agar plates containing tetracycline. After overnight incubation at 37 °C, the resulting CFUs in each plate were screened for GFP fluorescence, and were counted to calculate infection intensities. Four independent trials, each paired for both mosquito age (including stage) and *E. coli* dose, were conducted. Each trial consisted of approximately 10 mosquitoes per group, and the data were combined and analyzed using the Kruskal-Wallis test, followed by Dunn’s multiple comparisons *post-hoc* test.

### Quantification of circulating hemocytes

For each mosquito, an incision was made across the lateral and ventral portions of the suture that joins the 7th and 8th abdominal segment of naïve, injured (LB-injected), and *E. coli*-infected larvae, 1-day-old adults and 5-day-old adults at 24 h post-treatment. A finely pulled glass capillary needle was then inserted into the thorax, approximately 200 μl of Schneider’s *Drosophila* medium was perfused through the mosquito, and the diluted hemolymph was collected within two 1 cm diameter etched rings on a Rite-On glass slide (Gold Seal; Portsmouth, NH, USA) [46]. After allowing the hemocytes to adhere to the slide for 20 min, cells were fixed and stained using Hema 3 (Fisher Scientific, Pittsburgh, PA, USA), and, after drying, mounted with coverslips using Poly-Mount (Polysciences, Warrington, PA, USA) [45, 47]. The total number of hemocytes was then counted under bright-field conditions at 40× magnification using either a Nikon 90i compound microscope (Nikon, Tokyo, Japan) or an Olympus BH-2 microscope (Olympus, Tokyo, Japan). Three independent trials consisting of approximately 5 individuals per treatment group were conducted (*n* = 14–16 per group). Data were analyzed by two-way ANOVA, using age (including stage) and treatment as variables, followed by Šidák’s *post-hoc* test.

### Quantification of sessile hemocytes associated with the dorsal abdomen

The hemocytes from naïve, injured, and *E. coli*-challenged larvae, 1-day-old adults and 5-day-old adults were fluorescently labelled in vivo at 24 h post-treatment by injecting live animals with 67 μM of Vybrant CM-DiI Cell-Labeling Solution (Invitrogen, Carlsbad, CA, USA) and 1.08 mM Hoechst 33342 nuclear stain (350/461; Invitrogen) in PBS, and incubating them at 27 °C and 75% relative humidity for 20 min [11, 48]. Larvae were fixed for 5–10 min by immersion in 4% paraformaldehyde (Electron Microscopy Sciences, Hatfield, PA, USA) and then dissected along a coronal plane. Specimens were rinsed in PBS, and the dorsal abdomens, without the internal organs, were mounted on a glass slide using Aqua-Poly/Mount (Polysciences Inc., Warrington, PA, USA). Adults were fixed for 5–10 min by injection of 4% paraformaldehyde and then dissected along a coronal plane. Specimens were placed in 0.5% Tween in PBS, rinsed briefly in PBS, and the dorsal abdomens, without the internal organs, were mounted on a glass slide using Aqua-Poly/Mount.

Dissected larval and adult dorsal abdomens were imaged under bright-field and fluorescence illumination using a Nikon 90i microscope connected to a Nikon Digital Sight DS-Qi1 Mc monochrome digital camera and Nikon’s Advanced Research NIS Elements software. To render images with extended focal depth, Z-stacks of specimens were acquired using a linear encoded Z-motor, and all images within a stack were combined to form a single focused image using either NIS Elements’ Maximum Intensity Projection (for quantification) or Extended Depth of Focus (EDF; for viewing) modules.

Sessile hemocytes attached to the dorsal abdominal wall were quantified by two methods. First, custom polygonal regions of interest (ROI) were drawn on the maximum intensity projections to delineate the dorsal and lateral portions of abdominal segments 2–8, as was done previously for the periostial regions and tracheal tufts of larvae [11], and the mean pixel intensities of CM-DiI fluorescence within the ROIs were measured and recorded for each abdominal segment as well as for the entire dorsal and lateral abdomen (Additional file 1: Figure S1). For each specimen, background fluorescence was removed by subtracting the intensity values from the abdominal segments of each group’s corresponding unstained naïve specimens. Secondly, EDF images were used to manually count the sessile hemocytes within abdominal segments 4, 5 and 6, with these hemocytes categorized as being bound to one of three tissues: (i) the cuticular integument, (ii) the heart (periostial hemocytes), or (iii) the trachea. Data were analyzed by two-way ANOVA, using age (including stage) and treatment as variables, followed by Šidák’s *post-hoc* test.

### Quantification of phagocytosis by circulating hemocytes

Larvae, 1-day-old adults and 5-day-old adults were injected with *E. coli*, and 1 h later, hemocytes were perfused as described above, except that each mosquito was perfused with approximately 50 μl of Schneider’s medium and all the perfusate was collected onto a single etched ring. After allowing the hemocytes to adhere to the slide for 20 min, cells were fixed for 5 min by adding 50 μl of PBS containing 4% formaldehyde and 0.03 mM Hoechst 33342 nuclear stain. After removal of the fixation and staining solution, slides were mounted with coverslips using Aqua-Poly/Mount. Specimens were then examined under 100× magnification using simultaneous DIC and fluorescence illumination on a Nikon 90i microscope. For each mosquito, the number of bacteria that had been phagocytosed by each of the first 100 hemocytes viewed was counted. These values were used to calculate two parameters: (i) the phagocytic index, which is defined as the percentage of cells that engage in phagocytosis; and (ii) the phagocytic capacity, which is defined as the average number of phagocytosed bacteria per hemocyte [45]. Three independent trials consisting of 10 individuals per age were conducted. Data were analyzed by the Kruskal-Wallis test, followed by Dunn’s *post-hoc* test.

### Quantification of antimicrobial hemolymph activity

Using a hemolymph extraction method we adapted from a protocol used for *Drosophila* larvae and adults [49], hemolymph was extracted at 24 h after treatment from larvae, 1-day-old adults and 5-day-old adults that were naïve, injured, or treated with heat-killed *E. coli*, *M. luteus* or *Enterobacter* sp. Briefly, an incision approximately 5 mm in length was made into the bottom of a 0.6 ml microfuge tube using a feather blade, and freshly wounded larvae or adults were placed inside. This tube was then inserted into a 1.5 ml microfuge tube, which was centrifuged to collect the hemolymph. For each larval collection, up to ten individuals were placed on a Kimwipe to remove all external water before placing the larvae on the inner rim of the 0.6 ml tube. An insect pin was used to puncture the thorax of each larva and the tubes were centrifuged for 10 s in a mini centrifuge. This resulted in the collection of 1–2 μl of hemolymph. For each adult collection, 20–30 individuals were cold-anesthetized and an insect pin was used to puncture the lateral thorax. The mosquitoes were then placed in the 0.6 ml tube and centrifuged at 2152 rcf for 5 min at 4 °C. This resulted in the collection of 1–2 μl of hemolymph. Immediately after collection, hemolymph was stored in a -20 °C freezer until use.

Antimicrobial activity of hemolymph was measured via a zone of inhibition assay that we adapted from protocols developed for bumblebee workers [50] and mealworm beetle larvae [51]. Briefly, a 2 ml *M. luteus* culture was grown overnight in LB broth at 37 °C in a shaking incubator until it reached approximately OD_600_ = 10. The entire *M. luteus* culture was then added to 20 ml of sterile, liquid 1% LB agar that was maintained at 55 °C by immersion in a water bath. The solution was mixed, and 5 ml of the *M. luteus*-LB agar solution was poured into 9 cm diameter Petri dish plates. After solidifying, equidistant holes were created in the agar 1.5 cm from the outer edge of the Petri dish by piercing and suctioning using a 1 mm tip sterile glass Pasteur pipette. Each hole was then loaded with 1 μl of hemolymph and, once the hemolymph had been absorbed by the agar, the plates were placed upside down in a 37 °C incubator for 16 h. Plates were then scanned at 1200 pixels per inch using an Epson Perfection V600 scanner (Epson America, Inc., Long Beach, CA), and images were analyzed using ImageJ software [52]. For each sample, the diameters of the individual zones of bacterial growth inhibition were measured three times at different locations, and the average of these diameters was used to calculate the areas of the zones of inhibition. Five independent trials for each of the three bacterial treatments were conducted, each consisting of 2–3 larval and 1–2 adult pooled hemolymph samples per treatment group (naïve, injured, and treated with heat-killed bacteria; *n* = 12–15 and *n* = 5–6 for larval and adult groups, respectively). The data were combined and analyzed by two-way ANOVA, using age (including stage) and treatment as variables, followed by Šidák’s *post-hoc* test. These experiments were only conducted using plates seeded with *M. luteus* because, as we and others have found, the replication rate of *E. coli* in this in vitro assay outpaces the antimicrobial activity of hemolymph, thus precluding the measurement of zones of inhibition [51].

### Quantification of the phenoloxidase activity of hemolymph

Using the method described for the zone of inhibition assay, hemolymph was extracted from naïve, injured, and *E. coli*-infected larvae, 1-day-old adults and 5-day-old adults at 24 h post-treatment. Because we noted that using PBS as a solvent causes auto-oxidation of DOPA, and hence darkening that is not caused by phenoloxidase activity, all experiments utilized sterile water as the diluent, and not PBS. After adding 1 μl of extracted hemolymph to 50 μl of water, 10 μl of the diluted hemolymph solution (or water alone in the case of the negative control) was added to a cuvette containing 90 μl of one of the following three solutions: (i) 4 mg/ml 3,4-Dihydroxy-L-phenylalanine (L-Dopa; Sigma, St. Louis, MO, USA); (ii) 20 mg/ml Sodium Diethyldithiocarbamate Trihydrate (DETC; Fisher Scientific, Pittsburgh, PA, USA) and 4 mg/ml L-DOPA; or (iii) water alone. Absorbance readings were measured at 490 nm every 5 min for 30 min using a BioPhotometer plus spectrophotometer. Five independent trials were conducted, each consisting of 1 larval or adult sample per treatment group. The data were combined and analyzed by repeated measures two-way ANOVA, using age (including stage) and treatment as variables, followed by Šidák’s *post-hoc* test.

### Quantification of immunity gene expression by qPCR

Total RNA was extracted from the whole body of approximately forty larvae, twenty 1-day-old adults and twenty 5-day-old adults that were naïve, or had been injected 24 h earlier with LB (injury), or GFP-*E. coli*. RNA was purified after homogenization in TRIzol reagent (Invitrogen, Carlsbad, CA), and repurified using the PureLink Micro-to-Midi Total RNA Purification System (Invitrogen). For each sample, 5 μg of RNA was treated with RQ1 RNase-Free DNAse I (Promega, Madison, WI, USA) to remove any contaminating genomic DNA, and then used as template for cDNA synthesis using an Oligo(dT)_20_ primer and the SuperScript III First-Strand Synthesis System for RT-PCR (Invitrogen). Real-time quantitative PCR was performed using Power SYBR Green PCR Master Mix (Applied Biosystems, Foster City, CA) on a Bio Rad CFX Connect Real-Time PCR Detection System (Hercules, CA, USA). Relative quantification of mRNA levels was performed using the 2^-ΔΔC^_T_ method [53], and mRNA levels were calculated relative to the naïve groups of each mosquito age. In all experiments, ribosomal protein gene *RPS7* (AGAP010592) was used as the reference gene, and the ribosomal protein gene *RPS17* (AGAP004887) was used as a control. Primer sequences are listed in Additional file 2: Table S1. Melting curve analyses after RT-PCR confirmed that the cDNAs used in the experiments were free of genomic DNA contamination and that only the gene of interest was amplified. Three independent trials were conducted, and each trial was analyzed in duplicate. Data are presented as the average fold-change relative to the naïve group of a given life stage or adult age. Data for each gene were analyzed separately by age (stage) using the Kruskal-Wallis test, followed by Dunn’s *post-hoc* test.

## Results

### Larvae and newly-emerged adult mosquitoes kill bacteria in their hemocoels more efficiently than older adult mosquitoes

**Fig. 1:**
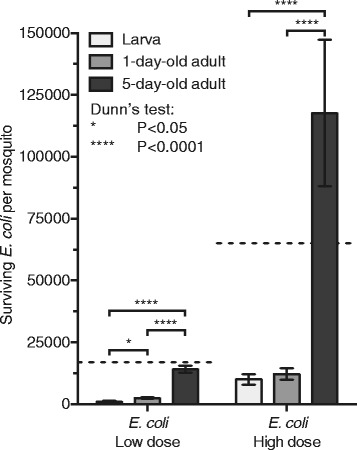
Average number of *E. coli* in the hemocoel (± SEM) of larvae, 1-day-old adults and 5-day-old adults at 24 h following infection with either a low dose (average = 18,061) or a high dose (average = 65,171) of *E. coli* (dotted lines indicate the doses). Data for each dose were analyzed by the Kruskal-Wallis test, followed by Dunn’s multiple comparisons *post-hoc* test

To begin to test whether larval and adult mosquitoes have different immune proficiencies, we infected larvae and differently aged adults with either low (OD_600_ = 1) or high (OD_600_ = 5) doses of *E. coli*, and measured their bacterial load 24 h later. Infections were conducted by means of intrahemocoelic injection to ensure that all animals received the same pathogen dose and to establish a precise time of infection. The 24 h time-point for sample collection was selected because it gave the mosquito ample time to combat the infection and allowed for the reading of larval immune parameters prior to pupation. It also allows for comparative analyses with other published works because 24 h is a commonly selected time-point. We found that, for both doses, bacterial killing proficiency differed significantly between the larval and adult stages (Fig. 1; OD_600_ = 1, Kruskal-Wallis: *H* = 62.85, *df* = 2, *P* < 0.0001; OD_600_ = 5, Kruskal-Wallis: *H* = 44.08, *df* = 2, *P* < 0.0001). Specifically, at 24 h following a low dose infection, the bacterial load in fourth-instar larvae, 1-day-old (newly-emerged) adults and 5-day-old adults had decreased by 94%, 86% and 22%, respectively. At 24 h following a high dose infection, bacterial load had decreased by 85 and 81% in fourth-instar larvae and newly-emerged adults, respectively, but increased by 81% in older adults. Statistical analysis of the data revealed that larvae killed significantly more bacteria than 1-day-old and 5-day-old adults following the low dose infection (Dunn’s test: *P* = 0.0179 and *P* < 0.0001, respectively), but when infected with the high dose, larvae were only more proficient at killing bacteria than 5-day-old adults (Dunn’s test: *P* < 0.0001). Taken together, these experiments show that larvae and 1-day-old adults display a stronger ability to combat systemic hemocoel infections than 5-day-old adults, and that the difference of the response is more pronounced at higher infection intensities.

### Larvae contain more circulating hemocytes than both newly-emerged and older adults, and larval hemocytes increase in abundance following infection

Because hemocytes are the first cellular responders to infection and kill pathogens via phagocytosis and the production of humoral immune factors [3, 54], we hypothesized that the developmental differences we observed in the killing of *E. coli* could be due to differences in the number of circulating hemocytes available to quell the infection. To test this, we counted the circulating hemocytes in naïve, injured (injected with sterile LB broth), and *E. coli*-infected larvae, 1-day-old adults and 5-day-old adults at 24 h after treatment.

**Fig. 2:**
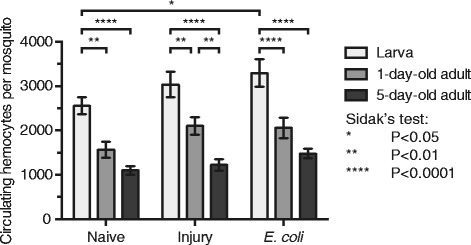
Average number of circulating hemocytes (± SEM) in naïve, injured, or *E. coli*-infected (high dose) larvae, 1-day-old adults, and 5-day-old adults at 24 h post-treatment. Data were analyzed by two-way ANOVA, followed by Šidák’s multiple comparisons *post*-*hoc* test

The number of circulating hemocytes changed significantly with mosquito life stage and age (Fig. 2; two-way ANOVA: *F*_(2, 126)_ = 51.73, *P* < 0.0001). Specifically, naïve larvae contain more circulating hemocytes (2553) than either 1-day-old adults (1561) or 5-day-old adults (1097), and a similar age-related pattern was observed for both injured and infected mosquitoes (Šidák’s multiple comparisons *post*-*hoc* test: *P* ≤ 0.0061 for all comparisons). Treatment also had a significant effect in the number of circulating hemocytes (two-way ANOVA: *F*_(2, 126)_ = 5.469, *P* = 0.0053), and this was due to an infection-induced increase in the number of circulating hemocytes in infected larvae compared to naïve larvae (Šidák’s: *P* = 0.0327), which is an effect that was not seen in either 1-day-old adults or 5-day-old adults (Šidák’s: *P* = 0.2492 and *P* = 0.4774, respectively). Together, these data show that the number of circulating hemocytes declines following the larva to adult transition, and that this number continues to decline as mosquitoes age.

### The spatial arrangement of sessile hemocytes differs between larvae and differently aged adults, and sessile hemocytes increase after infection in newly-emerged adults

We have previously shown that, regardless of the age of an adult mosquito, 75% of hemocytes circulate with the hemolymph whereas 25% are sessile, or attached to tissues [46]. Furthermore, the majority of sessile hemocytes are present on the dorsal abdominal wall or the tissues associated with it [46]. Thus, to compare the sessile hemocyte populations of larvae and adults, we labeled hemocytes in vivo using CM-DiI and quantified fluorescence intensity in the dorsal abdomen (Additional file 1: Figure S1).

**Fig. 3:**
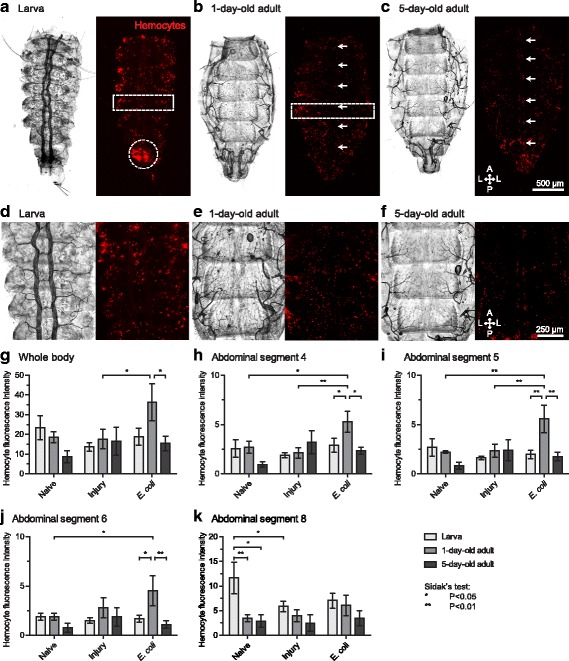
Sessile hemocyte arrangement and aggregation across life stages and in response to infection. **a**-**f** Dissected larva (**a**, **d**), 1-day-old adult (**b**, **e**) and 5-day-old adult (**c**, **f**) dorsal and lateral abdomens viewed under bright-field and fluorescence illumination at 24 h post-infection with *E. coli*. **d**-**f** show higher magnification images of segments 4, 5 and 6. Hemocytes were stained with CM-DiI (red). Larvae showed segmentally arranged hemocytes (box in **a**) as well as a high concentration of hemocytes in the 8th abdominal segment (circle in **a**). One-day-old adults showed an abundance of segmental hemocytes (box in **b**), including periostial hemocytes (arrows in **b**). Five-day-old adults displayed a similar arrangement except that segmental hemocytes were more dispersed, and periostial hemocytes were more distinct (arrows in **c**). *Directional arrows*: A, anterior; P, posterior; L, lateral. **g**-**k** Total fluorescence intensity of CM-DiI-stained hemocytes from the dorsal and lateral abdomen for abdominal segments 2–8 combined (**g**) or from abdominal segments 4 (**h**), 5 (**i**), 6 (**j**) and 8 (**k**) alone in naïve, injured, and infected larvae, 1-day-old adults and 5-day-old adults at 24 h post-treatment. Quantitative data were analyzed by two-way ANOVA, followed by Šidák’s *post*-*hoc* test. In **g**-**k**, whiskers denote the SEM. Data for abdominal segments 2, 3 and 7 are presented in Additional file 3: Fig. S2 and data for hemocyte counts in abdominal segments 4, 5 and 6 (**d**-**f**) are presented in Additional file 4: Fig. S3

Spatially, sessile hemocytes in larvae and 1-day-old adults are arranged in segmental bands that encircle each abdominal segment, but hemocytes do not retain this clear segmental pattern in 5-day-old adults due to fewer hemocytes present on the dorsal tergum (Fig. 3a-f). Furthermore, hemocytes in larvae are distinctly aggregated at the 8th segment tracheal tufts that surround the posterior opening of the heart (Fig. 3a), a phenotype not observed in adults. Instead, in adults, hemocytes are aggregated in the periostial regions of the heart (Fig. 3b–f), although this phenotype is less distinct in 1-day-old adults due to more surrounding hemocytes on the dorsal cuticle. Measurements of CM-DiI fluorescence intensity in the dorsal (tergum) and lateral (pleuron) surfaces of the abdomen did not detect differences in the abundance of sessile hemocytes between naïve and injured larvae, 1-day-old and 5-day-old adults (Fig. 3g). However, *E. coli* infection caused a significant increase in hemocyte intensity in 1-day-old adults relative to both injured 1-day-old adults and infected 5-day-old adults (Fig. 3g; Šidák’s: *P* = 0.0311 and *P* = 0.0220, respectively). When hemocyte intensity was analyzed for each abdominal segment (instead of the entire abdomen), we found that the infection-induced increase in sessile hemocytes of 1-day-old adults was due to strong increases in hemocyte intensity in segments 4, 5 and 6 (Fig. 3h-j), and to a lesser extent in the other abdominal segments (Additional file 3: Figure S2). In larvae, the fluorescence intensity of hemocytes was significantly elevated in the 8th abdominal segment, due to their strong association with the tracheal tufts, which is a phenotype not observed in adults (Fig. 3k).

To explore the abundance of sessile hemocytes on the dorsal and lateral abdominal wall in greater detail, we counted the number of hemocytes in segments 4, 5 and 6 and divided them by their anatomical location. We found that the infection-induced increase in sessile hemocytes in 1-day-old adults was due to an increase in the number of tracheal, cuticular, and periostial hemocytes relative to larvae and 5-day-old adults (Additional file 4: Figure S3; Šidák’s: *P* ≤ 0.0001 for all comparisons). Together, these results show that sessile hemocytes are numerous in larvae and adults, and that they increase in number after infection in newly-emerged adults. This infection-induced increase is particularly evident in the mid abdominal segments, which in adults is a region where there is high immune activity (for example, at the periostial regions) and high hemolymph flow [48, 55]. Furthermore, these findings confirm that the primary location of sessile hemocyte aggregation in larvae is at the tracheal tufts of the 8th abdominal segment, which are structures that surround the posterior openings of the larval heart [11].

### The phagocytic activity of individual hemocytes is higher in adult mosquitoes compared to larvae

**Fig. 4:**
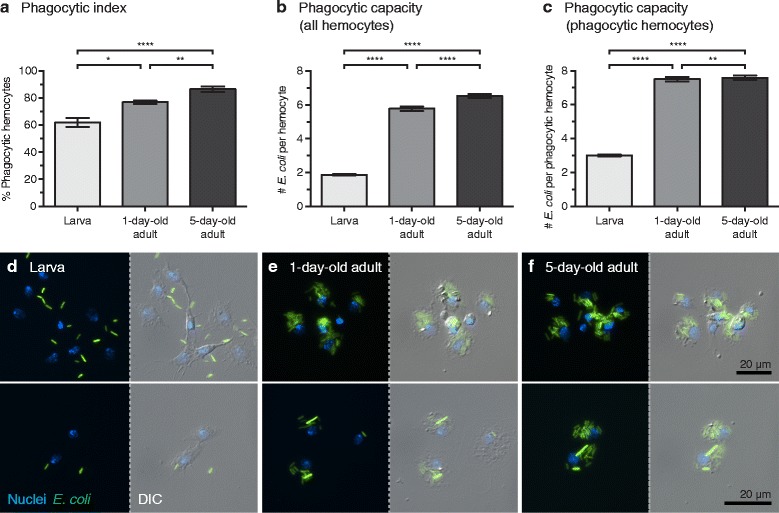
Hemocyte phagocytic burdens and spread in adults and larvae. **a** Percentage of larval and adult hemocytes that phagocytosed bacteria at 1 h post-infection with *E. coli* (phagocytic index). **b**, **c** Number of bacteria in individual larval or adult hemocytes at 1 h post-infection (phagocytic capacity). Data were analyzed for all hemocytes observed (**b**), and for only the hemocytes that had engaged in phagocytosis (**c**). Data were analyzed by the Kruskal-Wallis test, followed by Dunn’s *post-hoc* test (*P < 0.05, **P < 0.01, ****P < 0.0001). In **a**-**c** whiskers denote the SEM. **d**-**f** Larval and adult hemocytes viewed under both fluorescence and DIC illumination. Phagocytosed GFP-*E. coli* (green) is contained within hemocytes whose nuclei have been stained with Hoechst 33342 (blue). Many larval hemocytes display fibroblast-like morphology (**d**), whereas hemocytes from newly-emerged adults (**e**) and older adults (**f**) display a more rounded spreading morphology

Our initial hypothesis postulated that changes in the abundance of hemocytes underscored the increased resistance of larvae and newly-emerged adults to a bacterial infection. However, differences in immune proficiency may also arise from differences in the frequency or degree to which hemocytes engage in phagocytosis. To test phagocytic activity across life stages, larvae and adults were infected with GFP-*E. coli* and the phagocytic activity of circulating hemocytes was quantified 1 h later. The phagocytic index, defined as the percentage of hemocytes that engage in phagocytosis following an immune challenge [45, 47], differed significantly between larvae, 1-day-old adults, and 5-day-old adults (Fig. 4a; Kruskal-Wallis: *H* = 33.94, *df* = 2, *P* < 0.0001). Specifically, 60%, 75% and 85% of larval, 1-day-old adult and 5-day-old adult hemocytes phagocytosed bacteria at 1 h post-challenge, respectively (Dunn’s: *P* ≤ 0.0156 for all comparisons). Phagocytic capacity, defined as the number of bacteria internalized by individual hemocytes, also differed significantly (Fig. 4b; Kruskal-Wallis: *H* = 1319, *df* = 2, *P* < 0.0001), with larval, 1-day-old adult and 5-day-old adult hemocytes containing an average of 2, 6 and 7 bacteria per cell, respectively (Fig. 4b; Dunn’s *P* < 0 .0001 for all comparisons). When only hemocytes that phagocytosed bacteria were included in the analysis, a nearly identical pattern was observed (Fig. 4c; Kruskal-Wallis: *H* = 1031, *df* = 2, *P* < 0.0001; Dunn’s: *P* ≤ 0.0086 for all comparisons). Interestingly, in addition to differences in levels of phagocytosis, following infection many larval hemocytes displayed a fibroblast-like morphology with numerous filopodial extensions, which is different from the rounded morphology that is most often observed in adult hemocytes (Fig. 4d-f). Regardless of shape, these hemocytes are phagocytic suggesting that, similar to what we and others have found for adults [26, 56, 57], the vast majority of larval hemocytes are granulocytes. Together, these data show that, upon an identical bacterial challenge, the phagocytic burden of individual circulating hemocytes is higher in adults than it is in larvae (Fig. 4), although larvae contain more circulating hemocytes than adults (Fig. 2).

### Hemolymph from immune stimulated larvae has stronger antibacterial activity than hemolymph from adults

In response to infection, mosquitoes produce humoral immune factors that lyse pathogens [1, 7, 9, 10, 58]. To test whether the enhanced immunity we observed in mosquito larvae is correlated with enhanced humoral antimicrobial activity in their hemolymph, we employed a zone of inhibition assay to measure the general antibacterial activity of hemolymph.

**Fig. 5:**
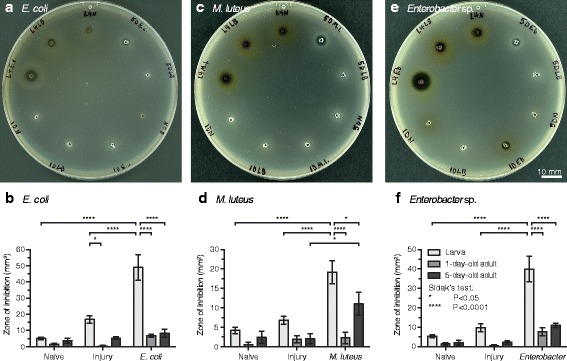
Larval and adult antibacterial humoral immunity. **a**, **b** Zone of inhibition plating assay (**a**), including quantitative measurement of the zones of inhibition (**b**), showing the extent of hemolymph lytic activity in mosquitoes that were naïve, injured, or challenged with heat-killed *E. coli* 24 h earlier. **c**-**f** Similar experiment except using heat-killed *M. luteus* (**c**, **d**) or heat-killed *Enterobacter* sp. (**e**, **f**) as the challenge agent. In **b**, **d**, and **f**, data were analyzed by two-way ANOVA, followed by Šidák’s *post*-*hoc* test. Whiskers denote the SEM. *Hand-written abbreviations on the plates*: L4, fourth-instar larvae; 1D, 1-day-old adult; 5D, 5-day-old adult; N, naïve; LB, injured; E.c., *E. coli*; M.l., *M. luteus*; Eb, *Enterobacter*

In trials involving heat-killed *E. coli*, the antibacterial activity of hemolymph differed significantly between larvae, 1-day-old and 5-day-old adults (Fig. 5a, b; two-way ANOVA: *F*_(2, 63)_ = 19.82, *P* < 0.0001). This was due to the significantly higher antibacterial activity of larval hemolymph compared to both adult groups (Šidák’s: *P* ≤ 0.0001 for both comparisons), and not to a difference between 1-day-old and 5-day-old adults (Šidák’s: *P* = 0.8717). Furthermore, this difference in antibacterial activity was due to an induced response, rather than a constitutive response, as the antibacterial activity in the hemolymph of naïve and injured individuals did not differ between larvae and adults, with the exception of when injured larvae were compared to 1-day-old adults (Šidák’s: *P* = 0.0387). To test whether this finding was unique to immune stimulation with Gram (−) *E. coli*, we repeated the experiment by stimulating mosquitoes with heat-killed Gram (+) *M. luteus* (Fig. 5c, d) and heat-killed Gram (−) *Enterobacter* sp. isolate Ag1 (Fig. 5e-f), a bacterial strain that naturally colonizes the midgut of *A. gambiae* and is a core adult bacterial symbiont taxon [43, 44]. In both sets of experiments, antibacterial activity of hemolymph was significantly higher in larvae compared to adults (Fig. 5d, F; two-way ANOVA: *F*_(2, 59)_ = 13.06, *P* < 0.0001 for *M. luteus* and *F*_(2, 65)_ = 13.17, *P* < 0.0001 for *Enterobacter* sp.; Šidák’s: *P* ≤ 0.0252 for all comparisons). This resulted from an induced response, as the antibacterial activity of hemolymph from naïve and injured mosquitoes did not differ between larvae and adults (Šidák’s: *P* ≥ 0.2856 for all comparisons). Although, based on these data it would be tempting to state that the immune system can discern between Gram (−) and Gram (+) bacteria because of the strength of the response, in this case it would be incorrect to do so because the doses and levels of pathogenicity are not equivalent across the different bacteria species. Together, these experiments show that the strength of humoral antibacterial activity of hemolymph is stronger in larvae compared to adults.

### Phenoloxidase activity is higher in larvae compared to adults

While conducting experiments measuring the antibacterial activity of hemolymph, we noticed that hemolymph, especially from larvae, had a tendency to darken rapidly after extraction, a phenomenon suggestive of the activation of phenoloxidase-mediated melanization. Because melanization is a major component of the humoral immune response of mosquitoes [59], we tested the relative strength of melanization in larval and adult hemolymph using a phenoloxidase activity assay [60, 61] that relies on the conversion of L-DOPA to dopachrome by phenoloxidase, a rate limiting enzyme in the melanization pathway [59, 62].

**Fig. 6:**
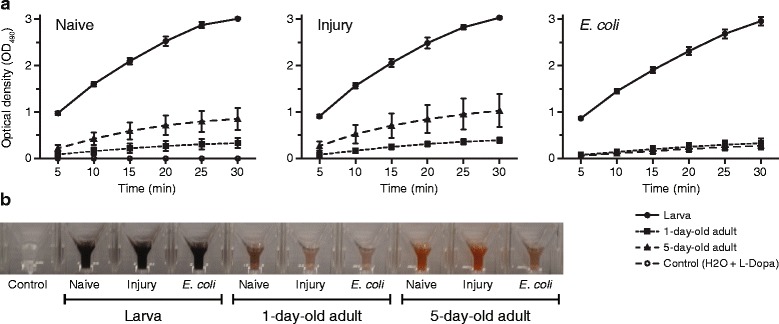
Hemolymph phenoloxidase activity of larvae and adults. **a** Time course of optical density (OD_490_) measurements of hemolymph from naïve, injured, and *E. coli*-infected larvae, 1-day-old adults, and 5-day-old adults that was diluted in water containing L-DOPA. Data were analyzed by two-way ANOVA, followed by Šidák’s multiple comparisons *post*-*hoc* test. Regardless of treatment and time point, larvae showed higher phenoloxidase activity than 1-day-old and 5-day-old adults (Šidák’s: *P* ≤ 0.0263 for all comparisons). Whiskers denote the SEM. **b** Images of cuvette wells at the 30 min time point showing that melanization-induced darkening is more pronounced in the larval samples

Measurements of the absorbance of diluted hemolymph (1:500 final dilution) incubated with L-DOPA, taken every 5 min for 30 min, showed that phenoloxidase activity differs significantly between life stages (Fig. 6a, b; repeated measures two-way ANOVA: *F*_(9, 40)_ = 62.99, *P* < 0.0001). Specifically, the phenoloxidase activity of larval hemolymph was significantly higher at all time points measured when compared to 1-day-old and 5-day-old adults (Šidák’s: *P* ≤ 0.0263 for all 5 min comparisons and *P* < 0.0001 for all 10–30 min comparisons). Phenoloxidase activity is a constitutive response, rather than an induced response, because no differences in phenoloxidase activity were detected between the hemolymph of naïve, injured and infected larvae or 1-day-old adults (Šidák’s: *P* > 0.9999 for all comparisons). In 5-day-old mosquitoes, infection reduced the melanization activity of hemolymph, with statistically significant differences beginning at the 20 min time point, suggesting that at the time of hemolymph collection the enzymatic cascade was partially depleted by immune responses during the ongoing infection (Šidák’s: *P* ≤ 0.0335 for all but two of six pairwise comparisons). No melanization activity was detected when L-DOPA was incubated in water in the absence of hemolymph, confirming that L-DOPA was not undergoing auto-oxidation (Fig. 6a; Additional file 5: Figure S4; Šidák’s: *P >* 0.9999 for all comparisons).

To quantify the melanization of endogenous substrates in diluted hemolymph, as opposed to the conversion of exogenous L-DOPA as in the above experiment, we repeated the experiment by diluting hemolymph samples in water alone, without L-DOPA. As expected, because of the absence of exogenous substrate, the absorbance values in all samples were very low (Additional file 5: Figure S4a). Nevertheless, larval hemolymph was significantly darker than adult hemolymph (Additional file 5: Figure S4b; Šidák’s: *P* ≤ 0.0086 for all comparisons), and no change in melanization occurred when comparing the initial and final readings, indicating that by the initial reading all endogenous substrates had been consumed in melanization reactions (Šidák’s: *P* ≥ 0.1690 for all comparisons).

Finally, to ensure that the darkening of hemolymph did not occur because of the activity of enzymes other than phenoloxidase, including iron-based peroxidase and catalase [59], we mixed diluted hemolymph with a solution containing both L-DOPA and diethyldithiocarbamate (DETC), a copper-specific chelator that inhibits copper-based phenoloxidase [59, 63–65]. Although larval hemolymph was again significantly darker than adult hemolymph because of the rapid melanization of endogenous (Additional file 5: Figure S4c, d; Šidák’s: *P* ≤ 0.0013 for all comparisons), melanization levels did not change following the addition of the solution of L-DOPA and DETC (Šidák’s: *P* ≥ 0.5840 for all comparisons), confirming that the melanization activity of hemolymph is driven by the activity of phenoloxidase. Together, these findings demonstrate that larval hemolymph contains greater melanization potential than adult hemolymph, and that this is due to the activity of phenoloxidase.

### Infection-induced transcriptional upregulation of immunity-related genes is higher in larvae than in adults

**Fig. 7:**
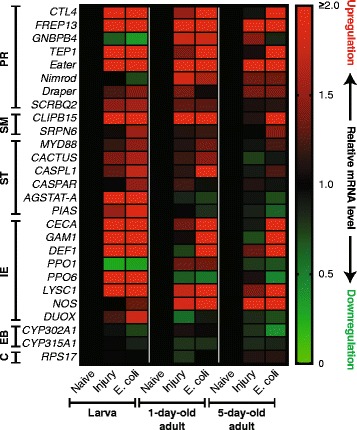
Infection-induced transcriptional regulation in larvae and adults. Heat map showing the average relative mRNA fold change of immunity-related genes in naïve, injured, and *E. coli*-infected larvae, 1-day-old adults and 5-day-old adults. Data are normalized against naïve individuals of the same age. Red color indicates upregulation, green indicates downregulation, and black indicates no regulation (see scale on right). For detailed graphs for each gene, see Additional file 6: Fig. S5 and Additional file 7: Fig. S6. *Abbreviations*: PR, pathogen recognition; SM, signal modulation; ST, signal transduction; IE, immune effectors; EB, ecdysteroid biosynthesis; C, control. In order, gene IDs from www.vectorbase.org are: AGAP005335, AGAP011197, AGAP002796, AGAP010815, AGAP012386, AGAP009762 (renamed AGAP029054), AGAP007256, AGAP010133, AGAP009844, AGAP009212, AGAP005252, AGAP007938, AGAP011693, AGAP006473, AGAP010423, AGAP005031, AGAP000693, AGAP008645, AGAP011294, AGAP002825, AGAP004977, AGAP007347, AGAP008255, AGAP009978, AGAP005992, AGAP000284 and AGAP004887

To determine whether larvae and differently aged adults differ with respect to the expression of immunity genes, relative mRNA fold change was assayed by qPCR at 24 h post-treatment in naïve, injured, and *E. coli*-infected larvae, 1-day-old adults and 5-day-old adults (Fig. 7; Additional file 6: Figure S5; Additional file 7: Figure S6; Additional file 2: Table S1). A broad range of immune responses was surveyed by measuring the transcription of 24 immunity genes representing four major categories of immune function: (i) pathogen recognition, specifically *CTL4* [66–68], *FREP13* [12, 69], *GNBPB4* [70], *TEP1* [71, 72], *Eater*, *Nimrod*, and *Draper* [73], and *SCRBQ2* [74, 75]; (ii) signal modulation, specifically *CLIPB15* [12, 22, 66] and *SRPN6* [76, 77]; (iii) signal transduction, specifically Toll pathway members *MYD88* and *CACTUS* [12, 78, 79], Imd pathway members *CASPL1* and *CASPAR* [80], and Jak/Stat pathway members *AGSTAT-A* and *PIAS* [14, 81, 82]; and (iv) effector responses, specifically *CECA* [12, 20, 83], *GAM1* [12, 21], *DEF1* [12, 28, 30, 84, 85], *PPO1* [12, 16, 86, 87], *PPO6* [16, 86], *LYSC1* [15, 88–90], *NOS* [91], and *DUOX* [92–95]. In addition, the expression of two genes involved in the ecdysteroid biosynthetic pathway, *CYP302A1* and *CYP315A1* [96, 97], was measured, as ecdysone peaks in the late larval and pupal stages and ecdysone induces the transcriptional upregulation of phenoloxidase genes in an *A. gambiae* hemocyte-like cell line [16, 87]. *RPS7* was used as the reference gene, and *RPS17* was used as a control [45].

In larvae, 17 immunity genes were either strongly (greater than 2-fold: 11) or weakly (between 1.5 and 2-fold: 6) upregulated by infection relative to naïve larvae, whereas in 1-day-old and 5-day-old adults, only 13 (11 strongly and 2 weakly) and 10 (9 strongly and 1 weakly) immunity genes were upregulated by infection, respectively, relative to naïve adults of their respective age (Fig. 7; Additional file 6: Figure S5; Additional file 7: Figure S6; Table 1). Transcription of ecdysteroid biosynthesis genes and, as expected, *RPS17* was not regulated by infection in any age group. In general, larvae and adults showed a similar level of infection-induced induction of some pathogen recognition and signal modulation genes, as well as some immune effector genes, such as cecropin (*CECA*), gambicin (*GAM1*), defensin (*DEF1*) and lysozyme (*LYSC1*). However, injury tended to result in a higher level of transcriptional induction in larvae when compared to adults.

**Table 1:** Summary of comparative immune data arising from experiments on larvae, 1-day-old adults, and 5-day-old adults. High, medium, and low responses are relative designations assigned by comparing each group to the other two groups and ranking them based on the strength of the response

	Larva	1-day-old adult	5-day-old adult
Bacterial killing	High	High	Low
Number of circulating hemocytes (increase after infection?)	High (Yes)	Medium (No)	Low (No)
Number of sessile hemocytes (increase after infection?)	Medium (No)	High (Yes)	Low (No)
Phagocytic burden	Low	High	High
Integrated immune and circulatory systems	Yes, at the tracheal tufts	Yes, at the periostial regions	Yes, at the periostial regions
Antimicrobial activity of hemolymph	High	Low	Low
Phenoloxidase activity of hemolymph	High	Low	Medium
Immune gene induction	High	Medium	Low
Genes transcriptionally upregulated > 2-fold (strongly)	*CTL4*, *FREP13*, *TEP1*, *Eater*, *CLIPB15*, *AGSTAT-A*, *CECA*, *GAM1*, *DEF1*, *PPO6*, *LYSC1*	*CTL4*, *FREP13*, *TEP1*, *Eater*, *CLIPB15*, *CASPL1*, *CECA*, *GAM1*, *DEF1*, *LYSC1*, *NOS*	*FREP13*, *TEP1*, *Eater*, *CLIPB15*, *CECA*, *GAM1*, *DEF1*, *LYSC1*, *NOS*
Genes transcriptionally upregulated 1.5–2-fold (weakly)	*SCRBQ2*, *SRPN6*, *CACTUS*, *CASPL1*, *PIAS, DUOX*	*GNBPB4*, *Nimrod*	*CTL4*
Genes transcriptionally downregulated < 0.5-fold (strongly)	*GNBPB4*, *PPO1*	None	*PPO6*, *CYP302A1*

When comparing the levels of induction, larvae showed greater than 2-fold higher induction (that is, the fold change of the larval group divided by the fold change of the adult group was >2) of *AGSTAT-A*, *PIAS*, *PPO6*, and dual oxidase (*DUOX*) relative to both adult groups, and this was due to infection causing transcriptional upregulation of these genes in larvae and weak transcriptional downregulation in adults. Both adult groups showed greater than 2-fold higher induction of nitric oxide synthase (*NOS*) and a fibrinogen-related protein gene (*FREP13*, also known as *FBN9*) relative to larvae, and 1-day-old adults showed greater than 2-fold higher induction of a CLIP domain serine protease (*CLIPB15*) relative to larvae, despite *FREP13* and *CLIPB15* being greater than 2-fold upregulated in response to infection in both life stages (Fig. 7; Additional file 6: Figure S5; Additional file 7: Figure S6).

In addition to differences in the level of induction of specific immunity genes across life stages, some genes were only upregulated in a single life stage. For example, infection in larvae, but not adults, induced the upregulation of Jak/Stat essential pathway genes *AGSTAT-A* and *PIAS*, as well as the immune effectors *PPO6* and *DUOX*. Conversely, infection in adults, but not larvae, strongly induced the upregulation of *NOS*. Interestingly, although the Gram (−) binding protein gene, *GNBPB4*, was transcriptionally downregulated in larvae following infection, when all the data were instead normalized against naïve 5-day-old adults, basal expression of *GNBPB4* in naïve larvae was 470- and 710-fold higher than in naïve 1-day-old and 5-day-old adults, respectively. A similar finding was made with respect to one of the phenoloxidase genes assayed, *PPO1*, which was expressed 170- and 110-fold higher in naïve larvae relative to naïve 1-day-old and 5-day-old adults, respectively. Collectively, these data show that induction of immunity genes differs with life stage and adult age, with infection-induced expression being highest in larvae and declining shortly after eclosion and as adults age.

## Discussion

Mosquitoes are susceptible to infections during all stages of their life cycle, and in response have evolved a robust innate immune system [1]. Mosquitos acquire pathogens via ingestion and through breaches in the outer cuticle, such as during injury. For example, larvae acquire *Bacillus thuringiensis* while feeding in their aquatic environment, and adults acquire *Plasmodium* sp. while imbibing infected vertebrate blood [10, 98]. Because the adult stage is responsible for the transmission of disease-causing pathogens such as malaria [9, 10], the vast majority of research on mosquito immunology has focused on that life stage, and in particular, on the adult females that feed on blood. In contrast, research on the immune system of the larvae of other insects, such as *Manduca sexta*, *Bombyx mori* and *Drosophila melanogaster* has proven invaluable for uncovering fundamental aspects of the cellular and humoral immune response of insects, and animals in general [99–101]. Nevertheless, lacking in Insecta are comparative studies that examine differences in immunity across the different life stages of a given species. As noted by Fellous & Lazzaro, “Almost all studies of the immune system of animals with metamorphosis have focused on either larval or on adult immunity, implicitly assuming that these traits are either perfectly correlated or evolutionarily independent” [41]. Because mosquito larvae live in aquatic environments rife with microorganisms and survival through the larval stages is required to reach sexual maturity, at the onset of this study we hypothesized that larvae have evolved more proficient means of neutralizing infections than adults. To compare larval and adult immunity in *A. gambiae*, we measured a broad range of immune parameters that were previously unexamined in larvae, and show that immune activity is strongest in larvae and wanes in the adult life stage (Table 1). Furthermore, because immunity phenotypes differ between life stages that are separated by metamorphosis, these findings suggest that adaptive decoupling, or the independent evolution of larval and adult traits made possible by metamorphosis [40], has occurred in *A. gambiae*.

Evidence for adaptive decoupling has been found in a broad range of animals with complex life cycles, including amphibians, fish, marine invertebrates and insects [40, 41, 102–108]. Several lines of evidence suggest that adaptive decoupling of immune responses has also occurred in the mosquito lineage. First, we have shown that the strength and composition of the mosquito immune response differs between immature larvae, newly-emerged adults and reproductively active older adults. Secondly, we have previously shown [11, 48, 55], and recapitulate here, that the mosquito circulatory and immune systems display stage-specific functional integration, enabling functionally analogous, yet anatomically disparate, immune strategies in larvae and adults. Thirdly, mosquitoes have complex life cycles with ecologically distinct larval and adult stages that are subject to different selection pressures, due to differences in pathogen exposure and reproductive status. Thus, we expect that a decoupling of key traits between these life stages, which are separated by metamorphosis, is advantageous to both stages in responding independently to differing selection pressures and in the performance of different fitness tasks (for example, food consumption and growth in larvae and reproduction in adults). Taken together, these arguments suggest that adaptive decoupling has occurred in mosquitoes, enabling the independent evolution of key larval and adult immune traits.

Our data show that larvae kill bacteria at a higher rate, possess more circulating hemocytes, have higher lytic and melanization activity in their hemolymph and have higher infection-induced expression of immunity genes than adults, thus strongly supporting our hypothesis that mosquito larvae invest more into immunity than adults. In our view this is not surprising; larvae inhabit bacteria-rich environments and display higher gut microbial diversity than adults [44, 109], suggesting that mosquitoes are exposed to a higher density and diversity of bacteria as larvae than as adults, and require more robust immune responses as larvae, as has been hypothesized previously [110]. Thus, given the vastly different ecologies between larvae and adults, coupled with the fact that evolutionary pressures are strongest on younger individuals that have yet to reach their reproductive potential [34, 35], we hypothesize that the stronger selection pressures at work in the larval stage, compared to the adult stage, have over time caused larvae to invest more greatly in immunity than adults.

Our findings on immune proficiency in larvae and adults is also indicative of immune senescence, as immunity in larvae is stronger than in adults, and immunity continues to weaken after metamorphosis, with 5-day-old adults mounting weaker immune responses than adults that were 4 days younger. This finding is consistent with well-attested evolutionary theories of senescence, which state that greater selection pressures are at work in the larval stage to ensure survival to the reproductively mature adult life stage [34, 36]. According to the “Disposable Soma” theory of aging, this occurs, in part, due to immune trade-offs that would not be in play at the larval stage, particularly those related to reproduction [111]. For example, resistance to pathogens in adult mosquitoes has been associated with reproductive costs [112–115]. Furthermore, numerous studies have documented age-associated declines in immune functions in mosquitoes [13, 26, 46, 47, 110, 116–120], fruit flies [121–123], butterflies [124, 125], bees [126–130], and other insects [131–135]. However, unlike these earlier studies, which focused almost exclusively on adults, our findings show that this trend extends into the earlier larval life stage. This suggests that mosquito adults employ a “live fast, die young” life history strategy [136], whereby immunity declines and mortality increases after adults reach reproductive maturity and selection for their survival wanes [34].

Although the data presented in this study conclusively show that larvae mount a stronger immune response than adults against bacteria that are present in the hemocoel, the adult stage has evolved mechanisms to boost the immune system when the possibility of infection is anticipated. Mainly, because many adult infections begin with an infectious blood meal [6, 10, 137], adult mosquitoes boost their immune reserves upon ingestion of blood. For example, in both mosquito subfamilies, acquisition of a blood meal induces the replication of hemocytes even in the absence of infection [138–140]. Whether this has a positive impact on the ability of anopheline mosquitoes to respond to an infection in the hemocoel remains unknown, but for *A. aegypti* (a culicine mosquito), blood feeding improves the ability to combat a low dose *E. coli* infection but is detrimental when facing a high dose *E. coli* infection [140].

The age-dependent decrease in immune proficiency correlates with age-related changes in the number of circulating hemocytes present in the hemocoel. We found that mosquito larvae contain more circulating hemocytes than adult mosquitoes, and that the number of larval hemocytes increases in response to infection. This is consistent with earlier studies conducted in *Anopheles gambiae* and *Aedes aegypti* adults that showed that immune stimulation or blood-feeding increases the number of circulating hemocytes [26, 45, 86, 138–142], and that the number of circulating hemocytes declines with adult age [26, 46, 47, 116]. Hemocytes are the first cellular responders to infection, phagocytosing pathogens in larvae and adults within minutes of infection [11, 48, 55, 57, 143, 144], and producing factors that limit both bacteria and *Plasmodium* infection [66, 86, 137, 145]. Thus, developmental changes in hemocyte profiles in part explain the higher bacteria killing efficiency of larvae relative to adults.

Although sessile hemocytes were abundant in both larvae and adults, the spatial arrangement of sessile hemocytes differed as adults aged, with newly-emerged adults displaying a pronounced segmental arrangement of hemocytes, which is more akin to what is observed in larvae than what is observed in 5-day-old adults [11, 46]. Furthermore, whereas hemocytes in adults aggregate at the periostial regions of the heart, hemocytes in larvae aggregate in the tracheal tufts of the 8th abdominal segment, where the sole incurrent openings into the heart is located. These differences are due to the stage-specific functional integration of the immune and circulatory systems, as hemocytes aggregate in the areas of highest hemolymph flow [11, 46, 48, 55]. These differences may also be explained by stage- and age-specific changes in heart physiology [42, 146]. Overall, these data show that sessile hemocyte populations undergo developmentally-related changes in spatial configuration and infection-induced abundance.

The finding that larvae have more circulating hemocytes than adults may account for the seemingly counterintuitive finding that larvae show lower phagocytic indices and capacities than adults. This is because larvae have more hemocytes available to phagocytose pathogens than adults, and based on our finding on the lytic and melanization activity of hemolymph, they also have a greater recourse to other means of pathogen clearance. Thus, an accumulation of phagocytic events in adults may signify a greater need for this immune mechanism, or lower phagocytic turnover rates, which has also been observed in aging fruit fly adults [121]. Interestingly, these differences in phagocytosis are accompanied by changes in hemocyte morphology. Larval hemocytes often had a fibroblast-like spread morphology, which has been observed in other insect hemocytes [27, 147], as opposed to the more rounded spread morphology typical of mosquito adult hemocytes [26, 46, 48, 56, 57, 143]. These fibroblast-shaped hemocytes have the functional characteristics of granulocytes, which are phagocytic, and not the functional characteristics of oenocytoids, which are the major producers of the phenoloxidase enzymes that drive the melanization pathway [3, 56, 57].

In addition to stage-specific differences in hemocyte biology, we detected profound differences in the melanization activity of hemolymph. Melanization is commonly used in the immune response against bacteria, malaria, and filarial worms [2, 7, 59, 148], and we found that mosquito larvae display a far greater capacity for phenoloxidase-mediated humoral melanization than adults. This finding is consistent with our recent observations of rapid and extensive melanin deposits in the abdomen of larvae following a bacterial infection [11], as well as with the decline in phenoloxidase activity that occurs with adult age in *Aedes aegypti* [117], and across life stage and with adult age in *Culex pipiens* [110]. In addition to enhancing humoral immunity, larvae may increase the melanization activity of hemolymph in preparation for cuticle tanning during and immediately after ecdysis, and for the rapid melanization of wounds in the more perilous aquatic environment of larvae.

In our final set of experiments, we show that immunity gene induction is generally stronger in larvae compared to adults, with the difference being exacerbated as adults age, and that larvae and adults differentially regulate the expression of some immunity genes. The higher rate of *E. coli* killing in larvae is consistent with the expression of immune effector genes such as the Gram (−) binding protein gene, *GNBPB4*, which was expressed 470- and 710-fold higher in naïve larvae compared to naïve 1-day-old and 5-day-old adults, respectively, as well as the higher expression of the phenoloxidase gene *PPO1*, which was expressed 170- and 110-fold higher in larvae relative to naïve 1-day-old and 5-day-old adults, respectively. The higher rate of melanization we observed in larval hemolymph is also consistent with the higher *PPO6* gene induction we observed in larvae, and supports our previous finding of higher infection-induced melanization in the abdomen of larvae when compared adults [11]. Furthermore, the higher melanization activity in the hemolymph of larvae is also consistent with the high level of dual oxidase (*DUOX*) gene induction detected in this life stage, as increased reactive oxygen species levels have been correlated with increased melanization [93]. In addition to the immune effector genes *PPO6* and *DUOX*, we also found that the Jak/Stat essential pathway member *AGSTAT-A* and, to a lesser extent, the negative regulator of this pathway, *PIAS*, were induced in response to *E. coli* infection in larvae and not in adults, showing that the same infection results in stage-specific induction of signaling pathway genes. The upregulation of Imd pathway member *CASPL1* in larvae and 1-day-old adults is consistent with this pathway’s role in combating Gram (−) bacterial infections, however the cause for Jak/Stat pathway involvement in the larval response alone is unknown, though it may have to do with its function in mosquito development [82], as this pathway is involved in *Drosophila* development [149]. Adults, not larvae, showed distinct upregulation of nitric oxide synthase, an important component of the adult immune response [91, 150], as well as higher upregulation of the pathogen recognition gene *FREP13* and the signal modulation gene *CLIPB15*. These differences in immunity gene induction across life stages could result from changes in the tissues expressing these genes during metamorphosis, as has been hypothesized previously [28, 41]. More broadly, however, these differences in immune gene regulation in larvae and adults are suggestive of adaptive decoupling, which would permit the independent regulation of larval and adult immune gene expression [40, 41].

Although the data presented herein show the formidable strength of the larval immune system relative to that of adults, these data were collected following the intrahemocoelic injection of a facultative pathogen, and not following feeding of an obligate pathogen. The route of administration and choice of pathogen was necessary to ensure that an identical dose with a known pathogen was provided to all groups, and to establish a precise time of infection. While many pathogens, including bacteria and entomopathogenic fungi, invade the hemocoel directly through the cuticle, future studies focusing on infections via the gut should be conducted to complement and refine our understanding of the present work. Furthermore, although this study did not track long-term mosquito survival (the larvae begin to pupate after 24 h), the long-term consequence of larval infection in the adult stage is an important gap in our current understanding and is an ongoing topic of study in our research group. Finally, our experiments on hemocyte biology did not differentiate between granulocytes, oenocytoids, or prohemocytes due to limitations inherent to the cell staining method, but it is well established that approximately 95% of hemocytes are granulocytes [3], and our qualitative observations suggest that life stage does not impact the proportion of hemocyte types.

## Conclusions

Studying larval immunity is crucial to our general understanding of mosquito biology, disease transmission, and vector control for several reasons. First, because mosquitoes are holometabolous insects, studying the larval immune system could yield important insights into how immune responses change over the course of development [11]. Secondly, larval environmental factors, including food availability, temperature, population density, competition and chemical insecticide exposure [151–159], as well as exposure to bacterial and fungal pesticides [98, 160–166], have all been shown to impact adult vector competence. Thirdly, many of the most widespread and effective mosquito control methods directly target the larval stages [167–169], and resistance to insecticides evolves more rapidly in larvae compared to adults, likely due to the stronger selection pressures at work in the larval stage [35]. The present study highlights both continuities and discontinuities between the larval and adult immune systems, and shows that mosquito larvae possess an enhanced immune system compared to adults. Hence, understanding the larval immune system of *Anopheles gambiae*, a major vector of malaria in Sub-Saharan Africa, could prove critical to the development and implementation of novel pest and disease control methods that are tailored to each life stage.

## Additional files

Additional file 1: Figure S1. Fluorescence emitted by sessile hemocytes in larvae and adults was measured using custom-drawn regions of interest. Representative images of dissected larva (**a**), 1-day-old adult (**b**) and 5-day-old adult (**c**) dorsal and lateral abdomens imaged under bright-field and fluorescence illumination at 24 h post-infection with *E. coli*. Hemocytes were stained with CM-DiI (red). Seven custom regions of interest (ROIs), encompassing abdominal segments 2–8, were used to quantify mean fluorescence intensity of CM-DiI stained hemocytes. ROIs delineated the dorsal (tergum) and lateral (pleuron) abdominal cuticle that lies between the abdominal sutures of adjoining abdominal segments. *Directional arrows*: A, anterior; P, posterior; L, lateral. (PDF 466 kb)

Additional file 2: Table S1. Gene names, VectorBase gene IDs, and primers used for quantitative RT-PCR. (PDF 30 kb)

Additional file 3: Figure S2. Sessile hemocytes are present in abdominal segments 2, 3 and 7. Fluorescence intensity of CM-DiI-stained hemocytes in abdominal segments 2 (**a**), 3 (**b**), and 7 (**c**) of naïve, injured, and *E. coli*-infected larvae, 1-day-old adults and 5-day-old adults at 24 h post-treatment. Quantitative data were analyzed by two-way ANOVA, followed by Šidák’s *post*-*hoc* test. Whiskers denote the SEM. Data for abdominal segments 4, 5, 6 and 8 are presented in Fig. 3. (PDF 116 kb)

Additional file 4: Figure S3. Sessile hemocytes in abdominal segments 4, 5 and 6 vary in response infection. Total number of CM-DiI-stained hemocytes attached to the trachea (**a**), cuticle (**b**, integument) and periostial regions of the heart (**c**) in abdominal segments 4, 5 and 6 of naïve, injured, and *E. coli*-infected larvae, 1-day-old adults and 5-day-old adults at 24 h post-treatment. Data were analyzed by two-way ANOVA, followed by Šidák’s *post*-*hoc* test. Whiskers denote the SEM. (PDF 122 kb)

Additional file 5: Figure S4. Endogenous melanization is highest in larvae, and exogenous melanization is completely inhibited by DETC. **a, b** Time course of optical density (OD_490_) measurements of hemolymph from naïve, injured, and *E. coli*-infected larvae, 1-day-old adults and 5-day-old adults diluted in water. The scale in **a** is amplified and separated by treatment in **b**. Larval hemolymph was significantly darker than adult hemolymph (Šidák’s: *P* ≤ 0.0086 for all comparisons) and melanization levels did not change when comparing the initial and final readings of any age or treatment group (Šidák’s: *P* ≥ 0.1690 for all comparisons). **c, d** Time course of optical density (OD_490_) measurements of hemolymph from naïve, injured, and *E. coli*-infected larvae, 1-day-old adults and 5-day-old adults diluted in saturated L-DOPA with DETC, which is a phenoloxidase inhibitor. The scale in **c** is amplified and separated by treatment in **d**. Larval hemolymph was significantly darker than adult hemolymph (Šidák’s: *P* ≤ 0.0013 for all comparisons) and melanization levels did not change from the initial to final readings of any age or treatment group (Šidák’s: *P* ≥ 0.5840 for all comparisons). Data were analyzed by two-way ANOVA, followed by Šidák’s *post*-*hoc* test. Whiskers denote the SEM. (PDF 138 kb)

Additional file 6: Figure S5. Relative expression of pathogen recognition and signal transduction genes. Graphs show the average mRNA fold change of pathogen recognition (**a**) and signal transduction (**b**) genes in naïve, injured, and *E. coli*-infected larvae, 1-day-old adults, and 5-day-old adults at 24 h post-treatment relative to the naïve group of a given life stage or adult age. Whiskers denote the SEM. Asterisks denote the significant regulation of mRNA levels relative to the naïve group as determined by the Kruskal-Wallis test, followed by Dunn’s *post*-*hoc* test (*P < 0.05, **P < 0.01, ***P < 0.001, ****P < 0.0001). (PDF 138 kb)

Additional file 7: Figure S6. Relative expression of signal modulation, immune effector, ecdysteroid biosynthesis, and ribosomal genes. Graphs show the average mRNA fold change of signal modulation (**a**), immune effector (**b**), ecdysteroid biosynthesis (**c**) and ribosomal (**d**) genes in naïve, injured, and *E. coli*-infected larvae, 1-day-old adults, and 5-day-old adults at 24 h post-treatment relative to the naïve group of a given life stage or adult age. Whiskers denote the SEM. Asterisks denote the significant regulation of mRNA levels relative to the naïve group as determined by the Kruskal-Wallis test, followed by Dunn’s *post*-*hoc* test (*P < 0.05, **P < 0.01, *** *P* < 0.001, ****P < 0.0001). (PDF 136 kb)

## Abbreviations

ANOVA: Analysis of variance
CFU: Colony forming unit
DETC: Sodium diethyldithiocarbamate trihydrate
DIC: Differential interference contrast
EDF: Extended depth of focus
GFP: Green fluorescent protein
LB: Luria-Bertani
L-DOPA: 3,4-Dihydroxy-L-phenylalanine
OD: Optical density
ROI: Region of interest
