# Supplementary material for: Anopheles gambiae larvae mount stronger immune responses against bacterial infection than adults: evidence of adaptive decoupling in mosquitoes
Source: Parasit Vectors. 2017 Aug 1;10:367. doi: 10.1186/s13071-017-2302-6 (PMC5539753; doi:10.1186/s13071-017-2302-6)
Supplement: Supplementary file 1 — Fluorescence emitted by sessile hemocytes in larvae and adults was measured using custom-drawn regions of interest. Representative images of dissected larva (a), 1-day-old adult (b) and 5-day-old adult (c) dorsal and lateral abdomens imaged under bright-field and fluorescence illumination at 24 h post-infection with E. coli. Hemocytes were stained with CM-DiI (red). Seven custom regions of interest (ROIs), encompassing abdominal segments 2–8, were used to quantify mean fluorescence intensity of CM-DiI stained hemocytes. ROIs delineated the dorsal (tergum) and lateral (pleuron) abdominal cuticle that lies between the abdominal sutures of adjoining abdominal segments. Directional arrows: A, anterior; P, posterior; L, lateral. (PDF 466 kb) [file 13071_2017_2302_MOESM1_ESM.pdf]

***Anopheles gambiae* larvae mount stronger immune responses against bacterial infection than adults: evidence of adaptive decoupling in mosquitoes**

Garrett P. League, Tania Y. Estévez-Lao, Yan Yan, Valeria A. Garcia-Lopez, and Julián F. Hillyer
Department of Biological Sciences, Vanderbilt University, Nashville, TN, U.S.A.
julian.hillyer@vanderbilt.edu

Parasites & Vectors, 2017

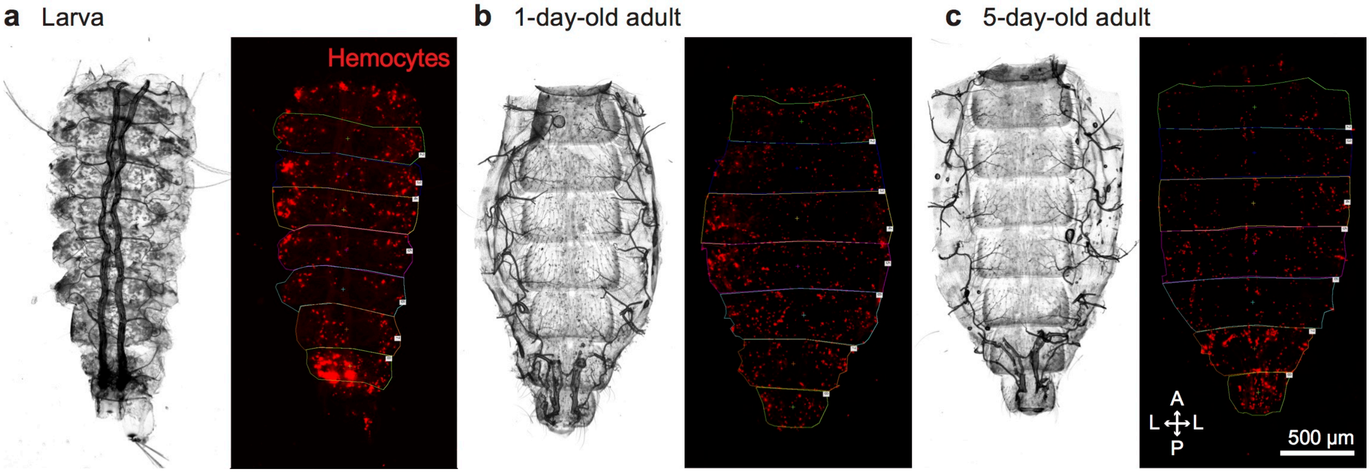

Additional file 1: Figure S1. Fluorescence emitted by sessile hemocytes in larvae and adults was measured using custom-drawn regions of interest. Representative images of dissected larva (**a**), 1-day-old adult (**b**) and 5-day-old adult (**c**) dorsal and lateral abdomens imaged under bright-field and fluorescence illumination at 24 h post-infection with *E. coli*. Hemocytes were stained with CM-DiI (red). Seven custom regions of interest (ROIs), encompassing abdominal segments 2–8, were used to quantify mean fluorescence intensity of CM-DiI stained hemocytes. ROIs delineated the dorsal (tergum) and lateral (pleuron) abdominal cuticle that lies between the abdominal sutures of adjoining abdominal segments. *Directional arrows*: A, anterior; P, posterior; L, lateral.
