# Supplementary material for: Anopheles gambiae larvae mount stronger immune responses against bacterial infection than adults: evidence of adaptive decoupling in mosquitoes
Source: Parasit Vectors. 2017 Aug 1;10:367. doi: 10.1186/s13071-017-2302-6 (PMC5539753; doi:10.1186/s13071-017-2302-6)
Supplement: Supplementary file 2 — Gene names, VectorBase gene IDs, and primers used for quantitative RT-PCR. (PDF 30 kb) [file 13071_2017_2302_MOESM2_ESM.pdf]

Additional file 2: Table S1. Gene names, VectorBase gene IDs, and primers used for quantitative RT-PCR.

Protein	Gene	VectorBase ID *	Assembly	Forward primer (5'→3')	Reverse primer (5'→3')	Amplicon (bp)	
						Transcript	Genomic
**Pathogen recognition**							
C-type lectin 4	*CTL4*	AGAP005335	AgamP4	TACCCAAAACCTGTGCGTTT	CTTTTCTGCGATCGTGTCAG	207	273
Fibrinogen-related protein 13	*FREP13*	AGAP011197	AgamP3	TTGTGATGAAGGAGCACAGC	GTGGTTGGAGCAGATGGTTT	176	176
Gram (-) binding pr. 4	*GNBPB4*	AGAP002796	AgamP3	TCAAGGACGGCATCTTCTTC	TCGGGTTGAGGTAGTTCGTT	174	250
Thioester-containing protein 1	*TEP1*	AGAP010815	AgamP4	GACGTCCAAATACGGATCTCA	CTTTCAGGCATCACCCGTAT	184	0
Eater	*Eater*	AGAP012386	AgamP3	TGGAGTCGACGAGAGGTTTC	TCGGTACGCATAAACCATCA	124	199
Nimrod **	*Nimrod*	AGAP009762	AgamP3	AACGAAGGGACACATTCCTG	CCGACTGAGAGATGGATTGG	121	179
Draper	*Draper*	AGAP007256	AgamP3	GTGACGAGCGTATGACGAAC	GCCGGTAGTACAGCAGCATT	132	1862
Croquemort	*SCRBQ2*	AGAP010133	AgamP3	AGGACGTGGCATTTTGTACC	TGGTTTGTTTTTCCACAGCA	177	281
**Signal modulation**							
CLIPB 15	*CLIPB15*	AGAP009844	AgamP3	CCGAACGATATCTGCATCCT	GCTCCACGTGCTTCTGTGTA	184	274
Serpin 6	*SRPN6*	AGAP009212	AgamP3	AGCGTATGCATCAGCGGTAT	TGGGCAGATCCTTCTGGTAG	195	279
**Signal Transduction**							
MyD88	*MYD88*	AGAP005252	AgamP4	ACCGCTGGTGTCTACCAATC	TTGCTCCCGCAGTAGAAACT	196	269
Cactus	*CACTUS*	AGAP007938	AgamP4	GGATGATTCCAGACGAGGAG	TCAGAAACTGCTGTGGAACG	204	318
Caspl1	*CASPL1*	AGAP011693	AgamP4	TGCTAGAAGCGCTGACCATA	GGGGCAAAAGTAGTCGATTG	217	217
Caspar	*CASPAR*	AGAP006473	AgamP4	CCGATCATCAATCAGCAGAA	GTGCAGATAGATCGCCAACA	204	647
Stat-A	*AGSTAT-A*	AGAP010423	AgamP4	CCTTGGAACAGGAAGAGCTG	ACGACCTACCCGTGCACTAA	218	218
Pias	*PIAS*	AGAP005031	AgamP4	ATCGACGTGCTCCCATCTAC	CCAGGAACCATCTTTGTGGA	205	294
**Immune effectors**							
Cecropin A	*CECA*	AGAP000693	AgamP3	GCTGAAGAAGCTGGGAAAGA	ATGTTAGCAGAGCCGTCGTC	158	247
Gambicin 1	*GAM1*	AGAP008645	AgamP3	TGCGAGATGTAAAAGCATCG	CAGCAGAGCTCCACTCAGAA	174	273
Defensin 1	*DEF1*	AGAP011294	AgamP3	GCAACGATCGTCTGTACCATT	GCTTGGCCCGATAGTTCTC	175	275
Phenoloxidase 1	*PPO1*	AGAP002825	AgamP4	CACCATCATGATCGAGAACG	GGAATTGTGACGGTGGATTC	207	296
Phenoloxidase 6	*PPO6*	AGAP004977	AgamP3	AGAGCCACTACCGGAAGGAT	TCGATGCTCTCAGCAATACG	174	242
Lysozyme C1	*LYSC1*	AGAP007347	AgamP3	ACGGCATCTTCCAGATCAAC	CATTGCAGTGGTTCTTCCAG	180	259
Nitric oxide synthase	*NOS*	AGAP008255	AgamP3	CAAGAGTGGGACCACATCAA	ACCCTTCTGGACCATCTCCT	129	210
Dual oxidase	*DUOX*	AGAP009978	AgamP3	TTTACCGGGCTAAAAGCAGT	ATTTGCGGCTCTTGTTCACT	175	251
**Ecdysteroid biosynthesis**							
CYP302A1	*CYP302A1*	AGAP005992	AgamP4	CATCCTGCCCACCGACCA	CCAAACGGAAGCACCAGGT	201	270
CYP315A1	*CYP315A1*	AGAP000284	AgamP4	CGAAGCGGAGTGCGTTGTT	CGTCTGTGCGTTTGCTATTC	202	274
**Ribosomal genes**							
Ribosomal pr. 17	*RPS17*	AGAP004887	AgamP3	GACGAAACCACTGCGTAACA	TGCTCCAGTGCTGAAACATC	153	264
Ribosomal pr. 7	*RPS7*	AGAP010592	AgamP3	GACGGATCCCAGCTGATAAA	GTTCTCTGGGAATTCGAACG	132	281

* VectorBase PEST strain genomic sequence (www.vectorbase.org) ** In AgamP4, AGAP009762 was renamed AGAP029054.

GP League, TY Estévez-Lao, Y Yan, VA Garcia-Lopez, and JF Hillyer. 2017. *Parasites & Vectors*
