# Supplementary material for: Anopheles gambiae larvae mount stronger immune responses against bacterial infection than adults: evidence of adaptive decoupling in mosquitoes
Source: Parasit Vectors. 2017 Aug 1;10:367. doi: 10.1186/s13071-017-2302-6 (PMC5539753; doi:10.1186/s13071-017-2302-6)
Supplement: Supplementary file 3 — Sessile hemocytes are present in abdominal segments 2, 3 and 7. Fluorescence intensity of CM-DiI-stained hemocytes in abdominal segments 2 (a), 3 (b), and 7 (c) of naïve, injured, and E. coli-infected larvae, 1-day-old adults and 5-day-old adults at 24 h post-treatment. Quantitative data were analyzed by two-way ANOVA, followed by Šidák’s post-hoc test. Whiskers denote the SEM. Data for abdominal segments 4, 5, 6 and 8 are presented in Fig. 3. (PDF 116 kb) [file 13071_2017_2302_MOESM3_ESM.pdf]

***Anopheles gambiae* larvae mount stronger immune responses against bacterial infection than adults: evidence of adaptive decoupling in mosquitoes**

Garrett P. League, Tania Y. Estévez-Lao, Yan Yan, Valeria A. Garcia-Lopez, and Julián F. Hillyer
Department of Biological Sciences, Vanderbilt University, Nashville, TN, U.S.A.
julian.hillyer@vanderbilt.edu

Parasites & Vectors, 2017

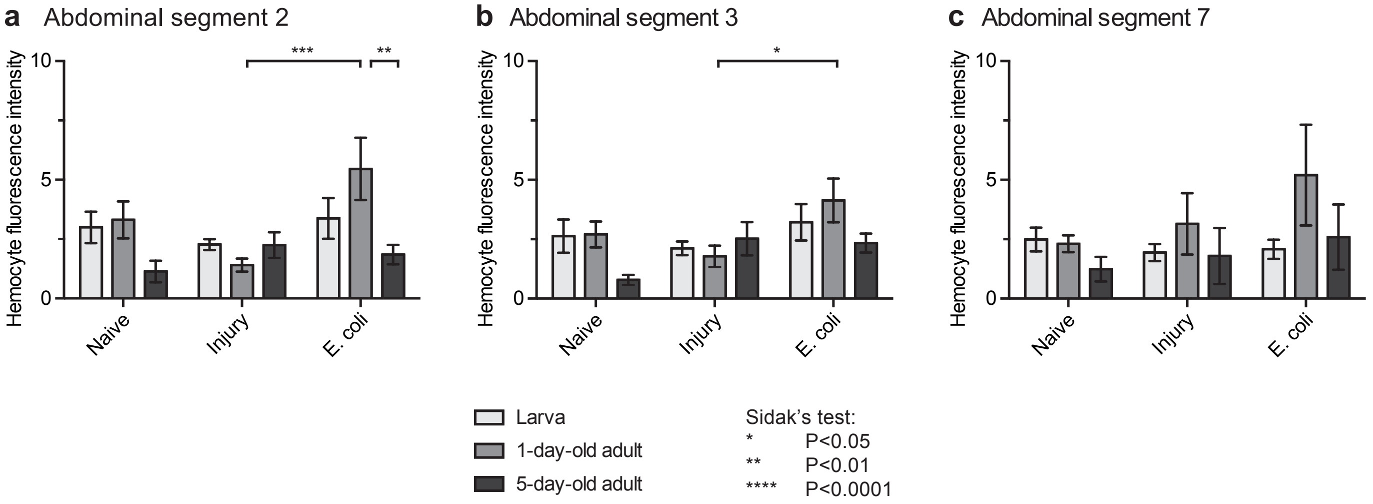

Additional file 3: Figure S2. Sessile hemocytes are present in abdominal segments 2, 3 and 7. Fluorescence intensity of CM-DiI-stained hemocytes in abdominal segments 2 (**a**), 3 (**b**), and 7 (**c**) of naïve, injured, and *E. coli*-infected larvae, 1-day-old adults and 5-day-old adults at 24 h post-treatment. Quantitative data were analyzed by two-way ANOVA, followed by Šidák's *post-hoc* test. Whiskers denote the SEM. Data for abdominal segments 4, 5, 6 and 8 are presented in Fig. 3.
