# Supplementary material for: Anopheles gambiae larvae mount stronger immune responses against bacterial infection than adults: evidence of adaptive decoupling in mosquitoes
Source: Parasit Vectors. 2017 Aug 1;10:367. doi: 10.1186/s13071-017-2302-6 (PMC5539753; doi:10.1186/s13071-017-2302-6)
Supplement: Supplementary file 4 — Sessile hemocytes in abdominal segments 4, 5 and 6 vary in response infection. Total number of CM-DiI-stained hemocytes attached to the trachea (a), cuticle (b, integument) and periostial regions of the heart (c) in abdominal segments 4, 5 and 6 of naïve, injured, and E. coli-infected larvae, 1-day-old adults and 5-day-old adults at 24 h post-treatment. Data were analyzed by two-way ANOVA, followed by Šidák’s post-hoc test. Whiskers denote the SEM. (PDF 122 kb) [file 13071_2017_2302_MOESM4_ESM.pdf]

Anopheles gambiae larvae mount stronger immune responses against bacterial infection than adults: evidence of adaptive decoupling in mosquitoes

Garrett P. League, Tania Y. Estévez-Lao, Yan Yan, Valeria A. Garcia-Lopez, and Julián F. Hillyer
Department of Biological Sciences, Vanderbilt University, Nashville, TN, U.S.A.
julian.hillyer@vanderbilt.edu

Parasites & Vectors, 2017

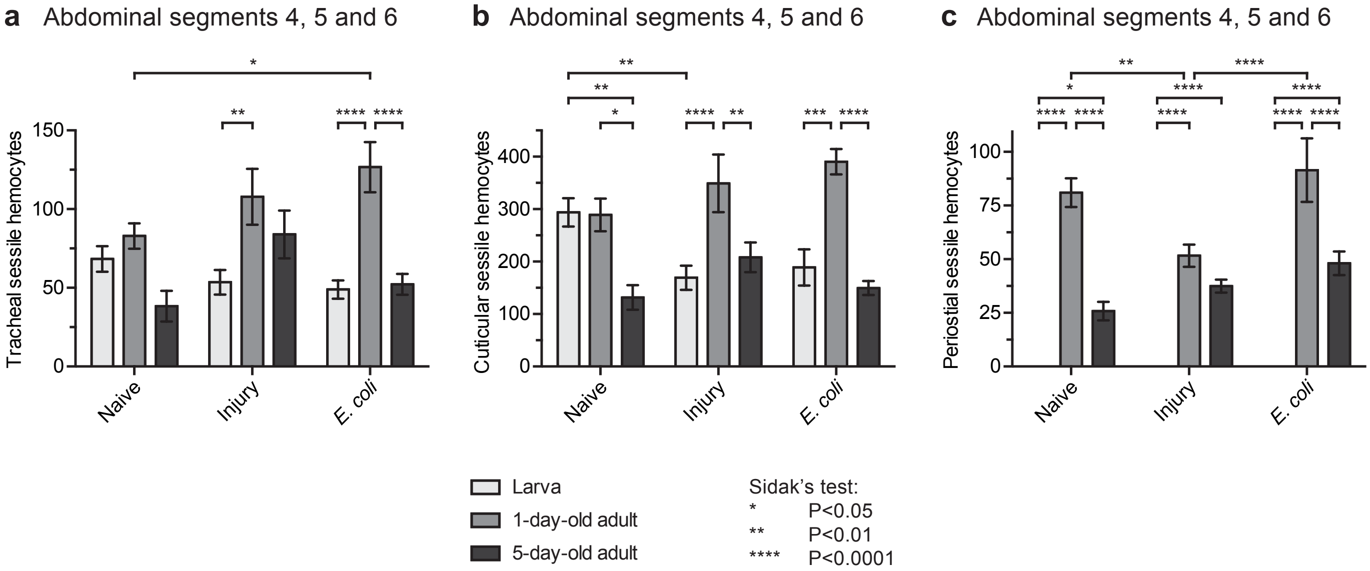

Additional file 4: Figure S3. Sessile hemocytes in abdominal segments 4, 5 and 6 vary in response infection. Total number of CM-DiI-stained hemocytes attached to the trachea (**a**), cuticle (**b**, integument) and periostial regions of the heart (**c**) in abdominal segments 4, 5 and 6 of naïve, injured, and *E. coli*-infected larvae, 1-day-old adults and 5-day-old adults at 24 h post-treatment. Data were analyzed by two-way ANOVA, followed by Šidák's *post-hoc* test. Whiskers denote the SEM.
