# Supplementary material for: Anopheles gambiae larvae mount stronger immune responses against bacterial infection than adults: evidence of adaptive decoupling in mosquitoes
Source: Parasit Vectors. 2017 Aug 1;10:367. doi: 10.1186/s13071-017-2302-6 (PMC5539753; doi:10.1186/s13071-017-2302-6)
Supplement: Supplementary file 5 — Endogenous melanization is highest in larvae, and exogenous melanization is completely inhibited by DETC. a, b Time course of optical density (OD490) measurements of hemolymph from naïve, injured, and E. coli-infected larvae, 1-day-old adults and 5-day-old adults diluted in water. The scale in a is amplified and separated by treatment in b. Larval hemolymph was significantly darker than adult hemolymph (Šidák’s: P ≤ 0.0086 for all comparisons) and melanization levels did not change when comparing the initial and final readings of any age or treatment group (Šidák’s: P ≥ 0.1690 for all comparisons). c, d Time course of optical density (OD490) measurements of hemolymph from naïve, injured, and E. coli-infected larvae, 1-day-old adults and 5-day-old adults diluted in saturated L-DOPA with DETC, which is a phenoloxidase inhibitor. The scale in c is amplified and separated by treatment in d. Larval hemolymph was significantly darker than adult hemolymph (Šidák’s: P ≤ 0.0013 for all comparisons) and melanization levels did not change from the initial to final readings of any age or treatment group (Šidák’s: P ≥ 0.5840 for all comparisons). Data were analyzed by two-way ANOVA, followed by Šidák’s post-hoc test. Whiskers denote the SEM. (PDF 138 kb) [file 13071_2017_2302_MOESM5_ESM.pdf]

Anopheles gambiae larvae mount stronger immune responses against bacterial infection than adults: evidence of adaptive decoupling in mosquitoes

Garrett P. League, Tania Y. Estévez-Lao, Yan Yan, Valeria A. Garcia-Lopez, and Julián F. Hillyer

Department of Biological Sciences, Vanderbilt University, Nashville, TN, U.S.A.

julian.hillyer@vanderbilt.edu

Parasites & Vectors, 2017

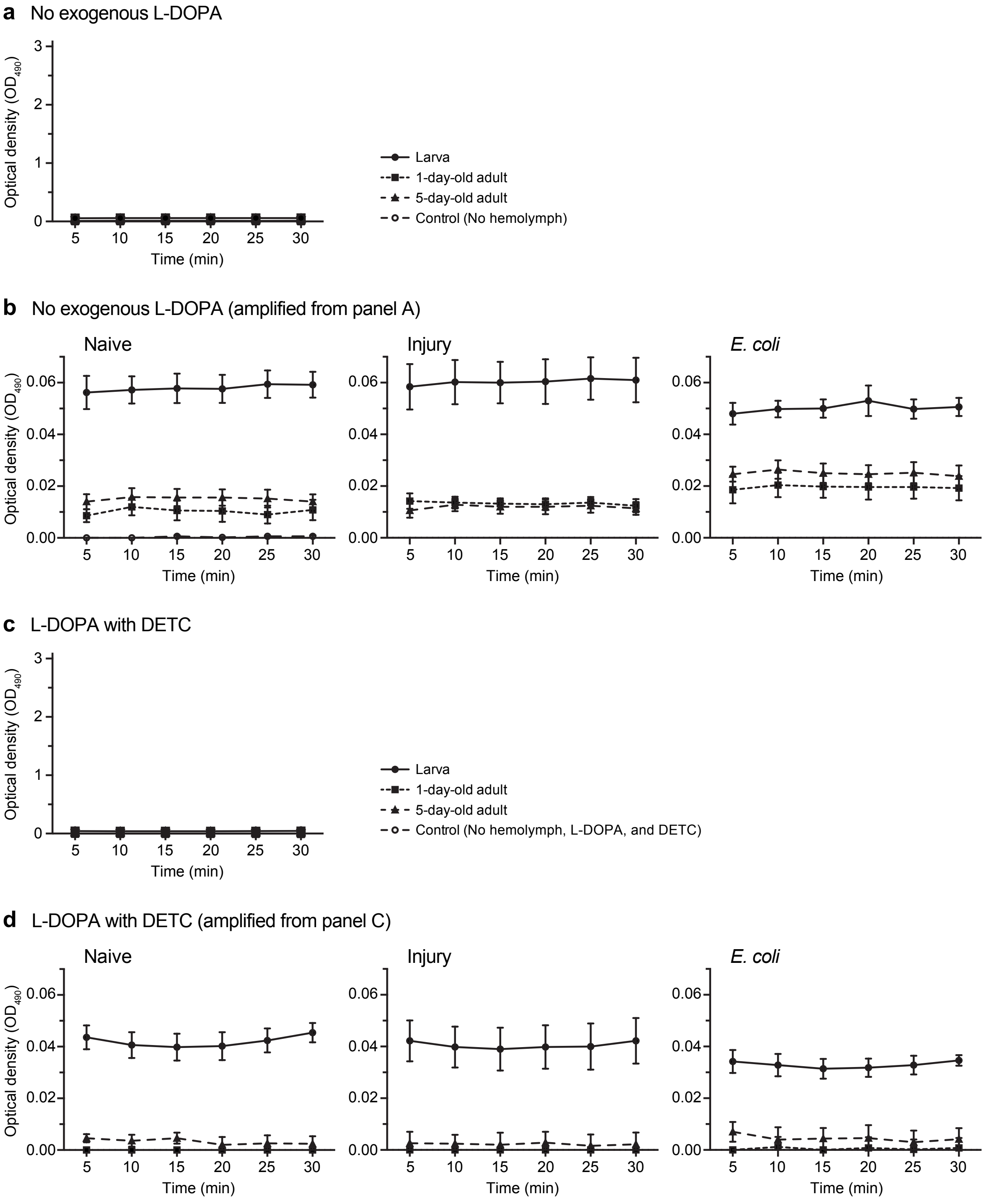

Additional file 5: Figure S4. Endogenous melanization is highest in larvae, and exogenous melanization is completely inhibited by DETC. **a, b** Time course of optical density (OD_{490}) measurements of hemolymph from naïve, injured, and *E. coli*-infected larvae, 1-day-old adults and 5-day-old adults diluted in water. The scale in **a** is amplified and separated by treatment in **b**. Larval hemolymph was significantly darker than adult hemolymph (Šidák's: $P \leq 0.0086$ for all comparisons) and melanization levels did not change when comparing the initial and final readings of any age or treatment group (Šidák's: $P \geq 0.1690$ for all comparisons). **c, d** Time course of optical density (OD_{490}) measurements of hemolymph from naïve, injured, and *E. coli*-infected larvae, 1-day-old adults and 5-day-old adults diluted in saturated L-DOPA with DETC, which is a phenoloxidase inhibitor. The scale in **c** is amplified and separated by treatment in **d**. Larval hemolymph was significantly darker than adult hemolymph (Šidák's: $P \leq 0.0013$ for all comparisons) and melanization levels did not change from the initial to final readings of any age or treatment group (Šidák's: $P \geq 0.5840$ for all comparisons). Data were analyzed by two-way ANOVA, followed by Šidák's *post-hoc* test. Whiskers denote the SEM.
