# Supplementary material for: Anopheles gambiae larvae mount stronger immune responses against bacterial infection than adults: evidence of adaptive decoupling in mosquitoes
Source: Parasit Vectors. 2017 Aug 1;10:367. doi: 10.1186/s13071-017-2302-6 (PMC5539753; doi:10.1186/s13071-017-2302-6)
Supplement: Supplementary file 7 — Relative expression of signal modulation, immune effector, ecdysteroid biosynthesis, and ribosomal genes. Graphs show the average mRNA fold change of signal modulation (a), immune effector (b), ecdysteroid biosynthesis (c) and ribosomal (d) genes in naïve, injured, and E. coli-infected larvae, 1-day-old adults, and 5-day-old adults at 24 h post-treatment relative to the naïve group of a given life stage or adult age. Whiskers denote the SEM. Asterisks denote the significant regulation of mRNA levels relative to the naïve group as determined by the Kruskal-Wallis test, followed by Dunn’s post-hoc test (*P < 0.05, **P < 0.01, *** P < 0.001, ****P < 0.0001). (PDF 136 kb) [file 13071_2017_2302_MOESM7_ESM.pdf]

Anopheles gambiae larvae mount stronger immune responses against bacterial infection than adults: evidence of adaptive decoupling in mosquitoes

Garrett P. League, Tania Y. Estévez-Lao, Yan Yan, Valeria A. Garcia-Lopez, and Julián F. Hillyer

Department of Biological Sciences, Vanderbilt University, Nashville, TN, U.S.A.

julian.hillyer@vanderbilt.edu

Parasites & Vectors, 2017

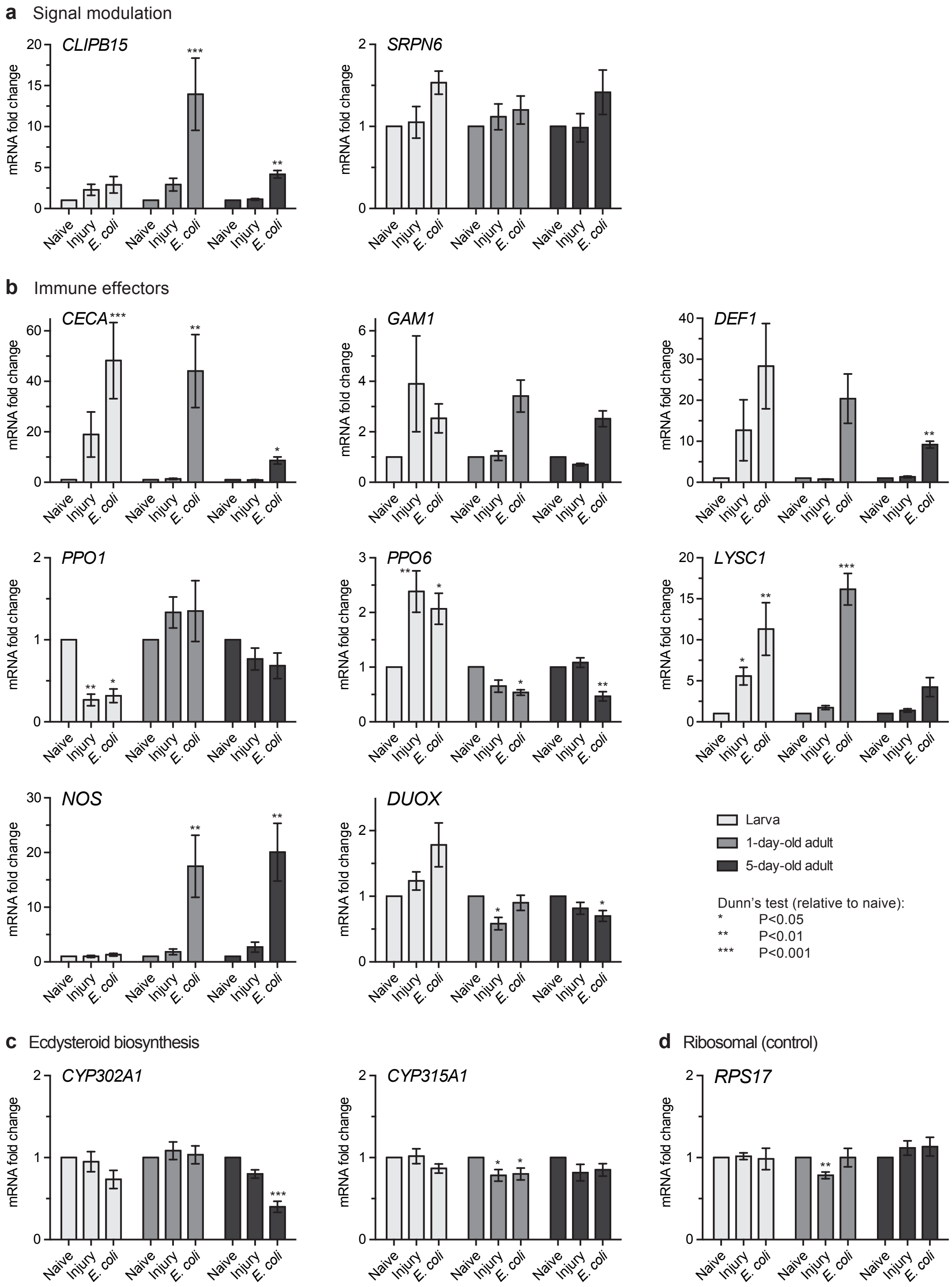

Additional file 7: Figure S6. Relative expression of signal modulation, immune effector, ecdysteroid biosynthesis, and ribosomal genes. Graphs show the average mRNA fold change of signal modulation (**a**), immune effector (**b**), ecdysteroid biosynthesis (**c**) and ribosomal (**d**) genes in naïve, injured, and *E. coli*-infected larvae, 1-day-old adults, and 5-day-old adults at 24 h post-treatment relative to the naïve group of a given life stage or adult age. Whiskers denote the SEM. Asterisks denote the significant regulation of mRNA levels relative to the naïve group as determined by the Kruskal-Wallis test, followed by Dunn's *post-hoc* test (*, $P < 0.05$; **, $P < 0.01$; ***, $P < 0.001$; ****, $P < 0.0001$).
